# Peripheral differentiation patterns of human T cells

**DOI:** 10.1002/eji.202149465

**Published:** 2022-03-30

**Authors:** Nelli Heikkilä, Iivo Hetemäki, Silja Sormunen, Helena Isoniemi, Eliisa Kekäläinen, Jari Saramäki, T. Petteri Arstila

**Affiliations:** ^1^ Translational Immunology Research Program (TRIMM), Research Programs Unit (RPU) Department of Bacteriology and Immunology, Medicum, Faculty of Medicine University of Helsinki Helsinki Finland; ^2^ Department of Computer Science Aalto University Espoo Finland; ^3^ Division of Transplantation and Liver Surgery Helsinki University Central Hospital Helsinki Finland; ^4^ Translational Immunology Research Program (TRIMM), Research Programs Unit (RPU) Department of Bacteriology and Immunology, Medicum, Faculty of Medicine HUSLAB Clinical Microbiology University of Helsinki, and Helsinki University Central Hospital Helsinki Finland

**Keywords:** T‐cell homeostasis, T‐cell memory, Recent thymic emigrant T cell, Lymphatic tissue

## Abstract

Long‐term T‐cell memory is dependent on the maintenance of memory T cells in the lymphoid tissues, and at the surface interfaces that provide entry routes for pathogens. However, much of the current information on human T‐cell memory is based on analyzing circulating T cells. Here, we have studied the distribution and age‐related changes of memory T‐cell subsets in samples from blood, mesenteric LNs, spleen, and ileum, obtained from donors ranging in age from 5 days to 67 years of age. Our data show that the main reservoir of polyclonal naive cells is found in the LNs, and the resting memory subsets capable of self‐renewal are also prominent there. In contrast, nondividing but functionally active memory subsets dominate the spleen, and especially the ileum. In general, the replacement of naive cells with memory subsets continues throughout our period of observation, with no apparent plateau. In conclusion, the analysis of lymphoid and nonlymphoid tissues reveals a dynamic pattern of changes distinct to each tissue, and with substantial differences between CD4^+^ and CD8^+^ compartments.

## Graphical abstract

We studied human T cells obtained from blood, spleen, ileal LNs, and ileal mucosa with flow cytometry and T‐cell receptor sequencing. Our results highlight the differences between CD4 and CD8 subsets in different tissues regarding their memory phenotypes and maturation trajectories.

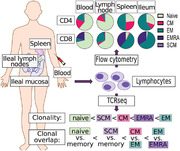

## Introduction

The adaptive immune system is characterized by its ability to mount a tailored, antigen‐specific response that can be rapidly reactivated at a new encounter with the same antigen, even decades after the initial antigenic exposure. In order to attain this end, the T‐cell repertoire must contain clonally diverse naive cells to respond to new threats and long‐lived memory cells to sustain immunity against the old. The process of memory generation has been shown to begin already during the fetal period and continues throughout the human lifespan [1, 2].

After the fetal period, all T cells develop in the thymus and exit it as recent thymic emigrant (RTE) T cells. RTE cells have been suggested to form a distinct population, which continues its maturation process in the periphery, giving rise to the antigen‐inexperienced, naive population [3]. Although declining sharply at the thymic involution after puberty, thymic activity continues well into adulthood, and new RTE cells replenish the naive population [4, 5, 6]. However, thymic activity again abruptly drops around 50 years of age, and thereafter the maintenance of T‐cell subsets is dependent on peripheral renewal driven by homeostatic signals [7]. The resultant decrease in the fraction of naive T cells may at some point affect the ability to respond to infections, explaining at least in part the age‐related impairment of immune defenses [8, 9]. Moreover, the remnants of active thymic tissue that have been observed even in centenarians may produce naive T cells of impaired fitness [10, 11, 12, 13, 14].

Upon antigen exposure, naive T cells differentiate into effector and memory subsets. Memory cells are characterized by clonal expansion and enhanced ability to respond to previously recognized antigens. Different memory T‐cell subsets exist with different characteristics: stem cell memory (SCM), central memory (CM), effector memory (EM) and terminal effector memory (EMRA) cells. A body of evidence suggests that these memory subsets arise from naive T cells in a progressive fashion naive→SCM→CM→EM→EMRA cells, although some level of plasticity also exists [15, 16, 17]. Two of these populations, SCM and CM, have been suggested to be responsible for the long‐term maintenance of memory responses. In particular, SCM cells exhibit clear proliferative capacity and can retain their phenotype during multiple rounds of proliferation, while also retaining the capability to differentiate to all the other memory subsets [16, 18, 19]. CM cells can also give rise to EM and EMRA cells, and to a limited extent they might also be able to generate SCM [20, 21]. In animal models, adoptive transfer of CM cells is enough to reconstitute protective immunity [22, 23]. In contrast, EM and EMRA cells possess the capability to rapid action, but only limited ability of self‐renewal [15, 21].

Several markers have been used to define the main T‐cell subsets. CD45 is a pan‐leukocyte marker with several isoforms of distinct expression patterns on T‐cell subsets. The long isoform CD45RA is expressed on naïve and SCM cells, absent on CM and EM and re‐expressed on EMRA cells [24]. The lymphoid chemokine receptor CCR7 is expressed by those subsets found predominantly in LNs and other organized lymphoid tissues [25]. The costimulatory molecules CD28 and CD27 are mostly expressed by quiescent subsets, while the loss of CD27 expression has been particularly linked to cellular senescence in EM cells [26]. The death receptor CD95 (FAS) is found on SCM cells but not on the other main subsets [16]. The combinations of markers used in our study to define the subsets are summarized in Table 1.

**Table 1:** Markers used to identify T‐cell subsets

	Naive	SCM	CM	EM	EMRA
CD45RA	+	+	−	−	+
CCR7	+	+	+	−	−
CD27	+	+	+	±	−
CD28	+	+	+	±	−
CD95	−	+			

Most studies on human T cells have analyzed circulating cells, but the great majority of memory T cells reside in other tissues, with major reservoirs in lymphoid tissues and associated with epithelia, in particular in the gut [27]. Although several recent publications have addressed the distribution of memory subsets outside the circulation, providing important insights of T cells in the tissues [28, 29, 30, 31, 32, 33], the maintenance of T‐cell memory in humans and the relationship between the different subsets remain incompletely understood [34, 35, 36].

## Results

### Distribution of recent thymic emigrants

**Figure 1:**
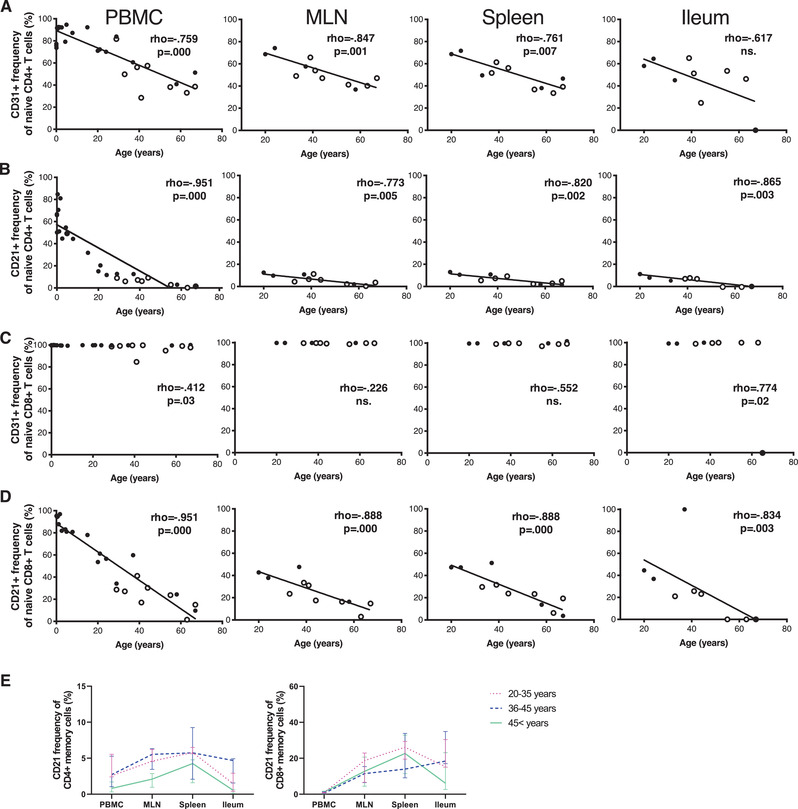
**CD31 and CD21 expression in T cells**. The expression CD31 (A) and CD21 (B) in naive CD4^+^ cells and the expression of CD31 (C) and CD21 (D) in naive CD8^+^ cells in PBMC, mesenteric lymph nodes (MLN), spleen, and ileum. The slopes represent Spearman's rho. The open circles represent CMV seropositive adult donors, and the black circles represent CMV seronegative adult donors and pediatric donors. The median frequency of CD21 in memory T cells in different age groups in different tissues (E). The bars display median and interquartile ranges. In A–D for PBMC, *n* = 27; for MLN and spleen, *n* = 11; for ileum, *n* = 9 in CD4^+^ and *n* = 10 in CD8^+^. In E for age group 20–35 years, *n* = 3 for all tissues; for age group 36–45 years, *n* = 4 except for spleen where *n* = 3; for >45 years, *n* = 5 for blood, *n* = 4 for MLN, *n* = 5 for spleen, and *n* = 3 for ileum. Samples from each individual were run as an independent experiment for a total number of experiments indicated.

T cells recently emigrated from the thymus form a distinct population, which continues its maturation process in the periphery [3]. The first marker used to identify the RTE population was CD31, but it has been mainly documented in the CD4^+^ compartment and more recent studies suggest it has a limited capacity to distinguish the RTE cells in the periphery [37]. We analyzed the fraction of CD31^+^ cells within the circulating naive T‐cell subset (*n* = 27), as well as in mesenteric LNs (*n* = 11), spleen (*n* = 11), and ileum (*n* = 9 for CD4^+^ and *n* = 10 for CD8^+^ T cells, gating strategy in Supporting Information Fig. S1). In all tissues, the expression of CD31 in CD4^+^ T cells showed an age‐dependent decrease. However, a substantial population of CD31 expressing naive CD4^+^ cells was present in all tissues even in the oldest donors in whom the thymic output should have ended (Fig. 1A). While CD31 expression seems to correlate with thymic output, it seems unreliable to distinguish the relative abundance of RTE cells and track their homing.

Recently, the complement receptor CD21 has been suggested to be a more accurate marker for RTE cells in both CD4^+^ and CD8^+^ subsets [38]. Supporting this, we observed a high frequency of CD21^+^ naive cells within the circulating CD4^+^ and CD8^+^ populations in the youngest children included in our study (Supporting Information Fig. S2). Expression of CD21 in naive T cells gradually declined with age and, in contrast to CD31, practically disappeared in naive CD4^+^ cells after the age of 50 years, consistent with the reported drop in thymic activity (Fig. 1B) [7]. In naive CD8^+^ cells, the difference between CD31 and CD21 expression was even more striking, with virtually all naive CD8^+^ cells expressing CD31 even in the oldest donors (Fig. 1C and D).

In blood, the expression of CD21 is enriched in naive T cells, but it has been reported that a small subset of IL‐8 secreting memory T cells can also express it [39, 40]. In our donors, the expression of CD21 in the circulating memory T cells was very low, but in other tissues CD21 was detected in antigen‐experienced T cells (Fig. 1E). Particularly in the spleen, a fraction of the CD8^+^ antigen‐experienced memory cells consistently expressed CD21 (median 22.7%; interquartile range 14.0–30.4%). In CD4^+^, but not in CD8^+^ memory T cells, the CD21 expression was higher in younger age groups than in the older, similarly to the naive CD4^+^ T cells (Fig. 1E).

RTE cells, defined as CD21^+^ naive T cells, were found in all tissues analyzed, particularly in the blood, and the next largest fraction was in LNs (Supporting Information Fig. S2). This was particularly clear in the CD8^+^ compartment. In our youngest organ donor, aged 20 years, 42.8% of naive CD8^+^ cells expressed CD21 and RTE cells accounted for 23.9% of CD8^+^ cells in the LNs (Fig. 1D and Supporting Information Fig. S2B). In spleen and ileum, both CD4^+^ and CD8^+^ RTE cells were less common and practically undetectable in individuals over the age of 50. In general, RTE cells were less frequent in CD4^+^ than in CD8^+^ compartment (Supporting Information Fig. S2).

### Subset distribution in blood

**Figure 2:**
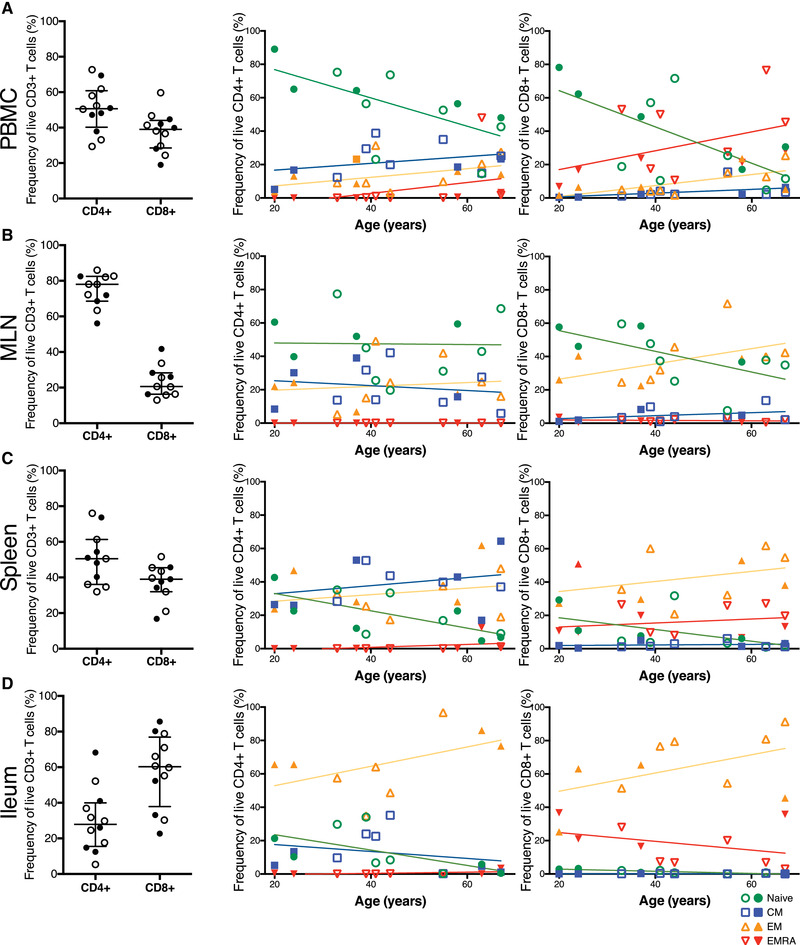
**Distribution of T‐cell subsets in tissues**. The plots show the frequencies of naive, CM, EM, and EMRA T‐cell subsets in the peripheral blood (A), MLN (B), spleen (C), and ileum (D) for CD4^+^ and CD8^+^ compartments. Naive, CM, EM, and EMRA subsets are indicated, respectively, with green circles, blue squares, yellow upward triangles, and red downward triangles. The open symbols represent the CMV seropositive and the closed symbols the CMV seronegative individuals. The slopes indicate the interpolation lines. In the subset distribution plots for PBMC, *n* = 12; for MLN and for spleen, *n* = 11; for ileum, *n* = 9 in CD4^+^ compartment and *n* = 10 in CD8^+^ compartment. Samples from each individual were run as an independent experiment for a total number of experiments indicated.

The current definitions of human memory T‐cell subsets are to a large extent based on circulating cells, adjusted more recently with analyses in other tissues. We based our definition on Gattinoni et al. [16]. In addition to CD45RA and CCR7, the naive, SCM and CM subsets were also defined by the expression of the costimulatory molecules CD27 and CD28, absent from EMRA and heterogeneously expressed in EM cells. CD95 expression was used to distinguish the SCM from the naive subset (Table 1). As previously reported, in both CD4^+^ and CD8^+^ population the largest circulating subset consisted of naive cells (median 41.1%; interquartile range 22.8–53.6%, and 15.5%; 9.5–58.4%, respectively), which both showed an age‐dependent replacement with antigen‐experienced memory subsets (Fig. 2A). While the decrease of naive cells was clearly observable, the increase of memory subsets was partly masked because the analysis was divided into different memory subsets with varying rates of change (Fig. 2). This replacement was particularly steep in CD8^+^ compartment, where the frequency of naive cells was below 30% in donors aged over 50 years. The distribution of memory subsets was also different between the CD4^+^ and CD8^+^ populations. In CD4^+^ T cells, the largest subset was formed by CM followed closely by EM cells. In contrast, the CD8^+^ memory compartment was dominated by EMRA cells, with clearly smaller fractions of EM and CM cells.

One female donor had a strikingly large population of circulating CD4^+^ EMRA cells (47.9%), whereas in other donors they formed a very small fraction. The same donor also had the highest frequency of circulating CD8^+^ EMRA cells (76%) in our cohort. Notably, she was one of the oldest donors (63 years) and a carrier of CMV, a particularly strong inducer of terminal T‐cell differentiation [41]. In mesenteric lymph nodes (MLN) and ileum from the same donor, the frequency of EMRA cells was comparable to the other elderly donors but in spleen their frequency was again elevated (12.7% vs. 1–2% in the other elderly donors).

### Subset distribution in peripheral tissues

Since the circulating T cells represent a transient population, a more thorough view of T‐cell homeostasis requires the analysis of tissue compartments. We therefore analyzed the distribution of T‐cell subsets in spleen, MLN and ileum as representative examples of both lymphoid and nonlymphoid locations. In the CD4^+^ compartment, the subset distribution in MLN most closely resembled the pattern in blood, with naive T cells forming the largest subset, followed by CM and EM cells (Fig. 2B). However, the age‐dependent replacement of naive cells with memory subsets was slower in MLN than in the blood. In the spleen, CM cells formed the largest subset, followed closely by the EM cells, while the frequency of naive cells was clearly lower (median 11.6%; interquartile range 4.4–21.8%) and decreased to <10% in the oldest donors (Fig. 2C). Ileum was dominated by EM cells (64.7%; 47.3–77.4%), which increased with age and reached a frequency of up to 95% in the oldest donors. Conversely, the resting populations, naive and CM, were small and the naive cells in particular seemed to vanish altogether with age (Fig. 2D).

In the CD8^+^ compartment, the distribution of subsets was again markedly different from that observed in the CD4^+^ compartment. In MLN, the CD8^+^ population was equally divided between naive and EM subsets (median 37.8%; interquartile range 30.8–50.5%, and 37.8%; 25.5–43.7%), while CM and EMRA cells were less frequent (Fig. 2B). Similarly to the CD4^+^ cells, the age‐dependent replacement of naive cells with memory subsets was slower in MLN than in the blood. In the spleen, the EM formed the largest CD8^+^ population (38.5%; 31.1–56.3%) followed by EMRA (13.3%; 9.7–25.9 %) and naive (3.3%; 1.5–10.2%) populations, although the frequency of naive cells diminished close to zero in the oldest donors (Fig. 2C). Likewise, the ileum was dominated by EM cells (64.8%; 48.6–81.4%) with a considerable fraction of EMRA cells as well (18.3%; 6.8–30.0%). Naive and CM populations were virtually absent in the ileal CD8^+^ compartment (Fig. 2D). Finally, the expression of T‐cell co‐stimulatory molecules CD27 and CD28 in EM cells varied markedly according to tissue and between CD4^+^ and CD8^+^ cells, mirroring the overall differences in CD27 and CD28 expression in these locations (Supporting Information Fig. S3). Loss of CD27 expression in particular is associated with T‐cell senescence, suggesting heterogeneity in proliferative capability within the EM subset [26].

We also analyzed the frequency of T‐cell subsets in the different tissues separately for those donors from whom we had a full set of samples with sufficient cell numbers (Supporting Information Fig. S4), showing the individual patterns of differentiation (*n* = 7 for CD4^+^ and *n* = 8 or CD8^+^ T cells).

### Distribution and age‐related changes in SCM T cells

**Figure 3:**
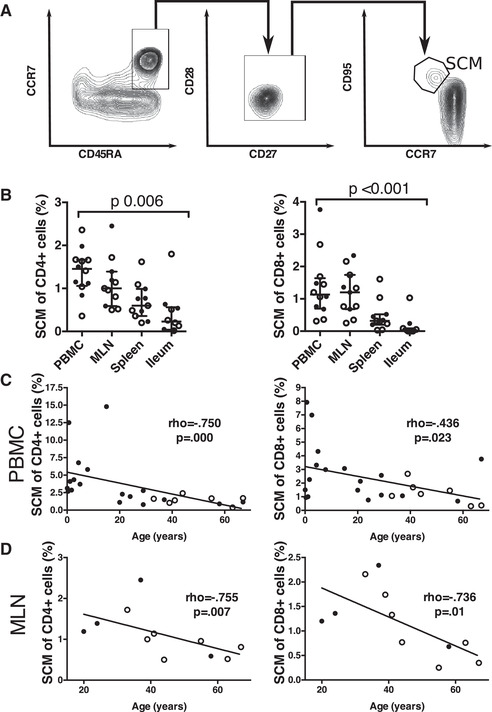
**The tissue distribution of SCM T cells**. The flow cytometric plots indicate the gating strategy of SCM cells (A). The diagrams show the frequency of SCM cells in CD4^+^ and CD8^+^ compartments in different tissues (B), the correlation of SCM frequency and age in CD4^+^ and CD8^+^ compartments in blood (C) and in MLN (D). The open circles represent CMV seropositive adult donors and the black circles represent CMV seronegative adult donors and pediatric donors. The comparison between groups is calculated with Kruskal–Wallis test (B). The slopes indicate the interpolation line for Spearmann's rho (C and D). In panel B f or PBMC, *n* = 12; for MLN and for spleen, *n* = 11; for ileum, *n* = 9 in CD4^+^ compartment and *n* = 10 in CD8^+^ compartment. Samples from each individual were run as an independent experiment for a total number of experiments indicated.

The recently identified SCM population has been suggested to play a crucial role in the long‐term maintenance of T‐cell memory [16, 42]. We therefore analyzed separately the tissue distribution and frequency of SCM cells in our donors (Fig. 3). In both the CD4^+^ and the CD8^+^ compartments, SCM cells were rare in all of the tissues studied, with the highest fractions found in the blood and MLN. In the spleen and the ileum, they were found only infrequently (Fig. 3B). Circulating SCM cells showed a strong age‐related decrease (Fig. 3C). MLN samples were not available from pediatric donors, but even in the adult donors the frequency of SCM cells in the MLN decreased with age (Fig. 3D). However, even in donors more than 50 years of age they formed a small but persistent population of CD4^+^ and CD8^+^ cells in both blood and MLN.

### Subset‐specific turnover rate

**Figure 4:**
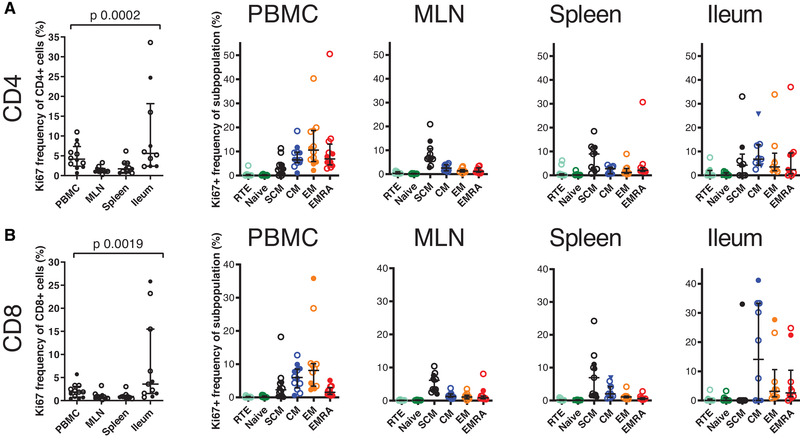
**Frequencies of proliferating T cells in different tissues**. The diagrams show the overall Ki67 expression and the Ki67 expression in different T cell subsets in different tissues for CD4^+^ (A) and CD8^+^ (B) compartments. The open circles represent the CMV seropositive and the closed circles represent the CMV seronegative individuals. The comparison between groups is calculated with Kruskal–Wallis test. For PBMC, *n* = 12; for MLN and spleen, *n* = 11; for ileum, *n* = 9 in CD4^+^ compartment and *n* = 10 in CD8^+^ compartment. Samples from each individual were run as an independent experiment for a total number of experiments indicated.

To analyze the maintenance of the T‐cell subsets, we measured the frequency of dividing cells, using the Ki67 cell cycle marker to identify them. Generally, the highest fractions of Ki67 expressing cells were detected in the ileum and the blood with considerably lower Ki67 expression in MLN and the spleen; this was true of both CD4^+^ and CD8^+^ cells (Fig. 4, Supporting Information Fig. S5). The turnover of naive T cells was very slow in all tissues, and expression of Ki67 in the CD21^+^ RTE fraction did not differ from that of other naive cells. The expression of Ki67 was clearly higher in all the memory subsets but showed considerable variation both between individuals and in different subsets and tissues. In tissues other than blood, EM and EMRA cells displayed a pattern of slow turnover, consistent with their limited capability of self‐renewal.

Notably, a relatively large fraction of CD4^+^ SCM cells expressed Ki67 in all tissues except blood. This was true also of MLN, in which the SCM population persisted with age. In the CD8^+^ SCM subset, the frequency of Ki67 expression was relatively high in MLN and the spleen but not in the blood. In ileum, the CD8^+^ SCM cells were so rare that the analysis of dividing cells should be interpreted with caution.

We also analyzed whether any of the subsets showed age‐dependent changes in the expression of Ki67, but no significant correlations were found (Supporting Information Fig. S6). Thus, the turnover rate seems to be intrinsic to the T‐cell subset and does not change during human lifespan.

### Diversity of T‐cell subsets in the MLN

To further study the T‐cell dynamics, we used TCR repertoire analysis to determine the clonal composition of the main subsets. Since LNs seemed to form an important long‐term reservoir of memory T cells, we isolated CD4^+^ naive, SCM, CM, and EM subsets from the MLNs of two donors (gating strategy in Supporting Information Fig. S7). We have recently shown that the TCRα chain is a more sensitive indicator of repertoire overlap than TCRβ, so we chose to sequence the TCRα repertoire of the isolated subsets [43, 44]. Because the size of the four subsets varied, the number of unique inframe clonotypes obtained was also different, ranging from an average of 424 in the SCM samples to an average of 38 000 in the naive samples (Table 2).

**Table 2:** Obtained numbers of total inframe TCRα sequences and unique inframe TCRα clonotypes

		CD4		CD8	
		Total inframe sequences	Unique inframe clonotypes	Total inframe sequences	Unique inframe clonotypes
**Naive**	**Donor 1**	55 815	42 064	38 737	27 632
	**Donor 2**	55 392	44 428	51 877	38 021
**SCM**	**Donor 1**	614	468	245	164
	**Donor 2**	815	629	582	433
**CM**	**Donor 1**	53 072	39 684	NA	NA
	**Donor 2**	39 019	25 385	NA	NA
**EM**	**Donor 1**	21 287	14 363	8 422	2 402
	**Donor 2**	43 183	30 973	45 789	10 631
**EMRA**	**Donor 1**	NA	NA	1 374	735
	**Donor 2**	NA	NA	14 437	4 537

**Figure 5:**
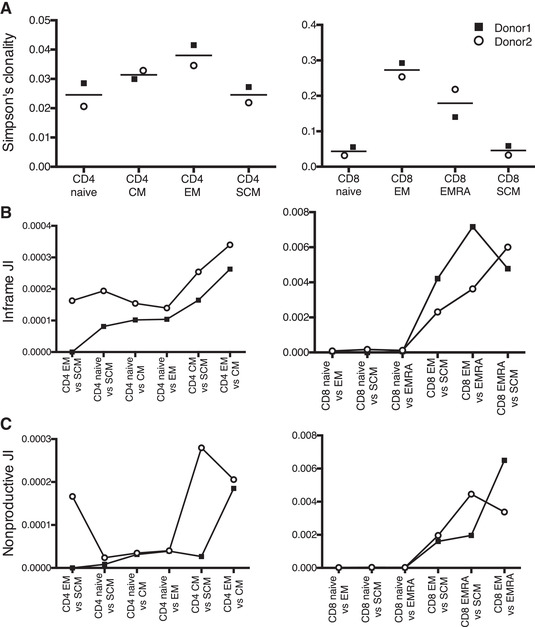
**TCRα clonotypes in naïve and memory T‐cell subsets**. The Simpson's clonality indexes in CD4^+^ naive, SCM, CM, and EM subsets and in CD8^+^ naive, SCM, EM, and EMRA subsets (A). The sharing of TCRα clonotypes between different T‐cell subsets for CD4^+^ and CD8^+^ compartments in the two donors assessed with Jaccard index (JI) for inframe TCRα repertoire (B) and nonproductive TCRα repertoire (C). Bars indicate the mean values (A). Squares indicate Donor 1 (CMV seronegative) and open circles indicate Donor 2 (CMV seropositive).

To compare the diversity of the CD4^+^ populations, we measured Simpson's clonality index for the inframe repertoires. To adjust for the different sample sizes in different populations, the analysis was performed with resampled datasets so that the larger populations (naive, CM, EM, EMRA) were subsampled to the number of unique clonotypes obtained in the smallest population (SCM) and the subsampling repeated 100 000 times. The average values of the subsamples were then used to compare the populations. Although only two donors were analyzed, the results were consistent. The highest clonality, indicating the presence of large clones and reduced diversity, was found in the EM cells, followed by CM cells, while naive and SCM cells were more polyclonal (Fig. 5A).

From the MLN CD8^+^ compartment, we isolated naive, SCM, EM, and EMRA subsets, and performed a subsampling to the size of the smallest population. For CD8^+^ cells also, the results were similar in both donors (Fig. 5A). The highest clonality was found in the EM cells, while EMRA cells, representing end‐stage differentiated cells, were surprisingly less clonal. Similarly to CD4^+^ cells, the naive and SCM cells were more polyclonal. Overall, the differences in clonality between memory subsets and naive cells were clearly higher in the CD8^+^ cells than in the CD4^+^ cells.

### Clonal overlap between T‐cell subsets in MLN

Finally, we measured the clonal overlap between the isolated subsets, again subsampling the larger repertoires to the size of the smallest sample. As a measure of overlap, we used the Jaccard index, calculated by dividing the number of shared clonotypes by the union of all clonotypes in the two samples. In the CD4^+^ compartment, the overlap between naive cells and memory subsets was generally lower than between the memory subsets (Fig. 5B). SCM cells had a higher fraction of shared sequences with CM than with EM cells, and clearly the highest overlap was detected between CM and EM cells. In the CD8^+^ compartment, the naive subset showed almost no overlap between any of the memory subsets. SCM cells were more similar to EM than to EMRA cells, and the highest overlap was between EM and EMRA cells (Fig. 5B).

We also analyzed the overlap in the nonproductive repertoire. Since the secondary, nonproductive rearrangements in the other TCRα locus do not result in an expressed protein, they are not subject to selection. Therefore, identical nonproductive sequences between two subsets suggest shared clonal origin. The patterns of overlap were largely similar to the productive repertoire, although there was some deviation in the analysis of CD4^+^ SCM cells (Fig. 5C).

## Discussion

Long‐term maintenance of effective immunological memory requires balancing between the need for self‐renewal and prolonged survival, on the one hand, and the capability for rapid function, on the other hand. The immune system solves this by the heterogeneity of memory T‐cell population. Our results confirm and expand previous studies from animal models and human samples [34, 45, 46], showing the predominance of functionally active, non‐dividing memory subsets at the ileal mucosa. In lymphoid tissues, the subset distribution is more evenly divided between resting CM cells and EM and EMRA cells. Our results also provide further support for the view that memory is formed differently in the CD4^+^ and CD8^+^ compartment, with very small numbers of CM cells and much larger fractions of functionally active EM and EMRA cells in the latter population [28]. The difference in TCRα clonality between naive and memory subsets was also more pronounced in the CD8^+^ compartment. This has previously been shown for TCRβ CDR3 repertoires in naive and EM populations, and together with our TCRα data suggests larger clonal expansions in the CD8^+^ than in the CD4^+^ population [7]. Finally, our data indicate that the complement receptor CD21, previously used to identify CD4^+^ RTE cells, can also identify CD8^+^ RTE cells when used together with markers identifying naive cells. This is consistent with Pekalski et al., who reported that CD21^+^ CD4^+^ cells have higher T‐cell excision circle content than their CD21– counterparts, and that the depletion of T cells is followed by an influx of CD21^+^ T cells, as would be expected for a RTE marker [38]. Thus, we were able to analyze the tissue distribution and age‐related changes of both CD4^+^ and CD8^+^ RTE cells. However, we also found CD21 expression on a subset of memory cells. This suggests that this complement receptor may have a functional role in both RTE cells and in certain memory subsets, especially in CD8^+^ memory T cells.

Analysis of the age‐related changes in the distribution of T‐cell subsets shows that the replacement of naive cells with memory cells is a life‐long process, with no apparent plateau in any of the tissues analyzed. Second, the turnover rate within any given subset does not change with age, but rather seems to be intrinsic to the subset in question though some cell populations were so small that the interpretation of Ki67 expression in these needs to be done cautiously. The highest turnover was found in the SCM and CM subsets, consistent with their self‐renewal capability. Recently, it was suggested that the originally described SCM population consists of two distinct subpopulations, one with a half‐life of less than 1 year and another with almost a decade [47]. Our data regarding the SCM population do not allow us to directly address this putative heterogeneity. However, our analysis of clonality shows that in their main reservoir, LNs, the SCM cells are more polyclonal than EM or EMRA cells, which may indicate the absence of significant clonal expansions, such as would be associated with substantially different turnover rates within the population.

Together with earlier reports [28, 29, 30, 31, 32], our data indicate that different tissues contribute in distinct ways to the homeostasis of human T‐cell populations. Thymic activity feeds RTE cells to the peripheral organs where they gain mature naive phenotype within 2–3 weeks, and in the younger donors RTEs are found at a low frequency in all tissues but especially in LNs [4, 28]. So far there has not been a way to identify CD8^+^ RTE cells, but our data suggest that when used within the naive population, CD21 is a reliable marker for them, as well. Similarly to CD4^+^ RTE cells, CD8^+^ RTE cells are enriched in LNs, and both subsets show an age‐related decrease, seen both in the frequency of RTE cells and in their fraction within the naive population. Since RTE cells and other naive cells had a similar turnover rate, this decrease likely reflects the age‐related changes in thymic function. At the same time, the naive cells themselves are slowly replaced by antigen‐experienced memory subsets, although they persist at substantial numbers in peripheral tissues up to the end of our age range. The slow homeostatic proliferation of naive cells in circulation has been shown to maintain a reservoir of polyclonal cells capable of reacting to novel antigens [48]. Our data indicate that the homeostatic maintenance of the naive population takes place mainly in the LNs.

While the maintenance of naive T cells provides precursors for novel responses, the functionality of the peripheral T‐cell populations depends on the long‐term maintenance of memory. For both CD4^+^ and CD8^+^ cells, the small subset of SCM cells has been implicated in the maintenance of the long‐term memory, and their numbers have been reported to remain stable, when analyzed in blood samples taken from individuals aged from 23 to 85 years [49]. In contrast, our data show a clear age‐related decrease in the number of SCM cells in the peripheral blood, as well as in the LN. However, after the steep decrease of SCM numbers during childhood, the rate of decrease in adulthood was clearly slower, and SCM cells were present in blood and particularly in LNs of even the oldest donors. In nonhuman primates, the SCM cells have been shown to form a life‐long memory T‐cell reservoir [50]. In addition to the SCM population, our data are consistent with a previous suggestion that CD4^+^ memory T cells are also maintained in the LNs by the CM population. For the CD8^+^ cells, the EM population seemed to be more important [45]. Notably, all memory subsets showed a larger clonal overlap with other memory cells than with naive cells, suggesting that they have been formed by the same antigen exposures. The higher overlap between the resting and functionally active memory subsets, in comparison to the overlap between the naïve and memory subsets, is also consistent with a scenario in which the former is responsible for the replenishment of the latter, nondividing subsets.

A caveat of our data is that some of the populations we analyzed have a very low frequency, and variation between the individuals was considerable. Similar variation has been, however, a consistent finding in other studies of human donors, and therefore is likely to reflect the biological reality [28, 29, 51]. Also, in our analysis of TCR repertoire only two donors were included, so the conclusions must remain tentative. Even so, in both donors the data were similar in broad outlines.

How then is new memory incorporated and what happens with the old? The fact that the ratio between memory and naive T cells grows throughout the human lifespan may reflect the simple possibility in which memory cells specific to new antigens are simply added to the pre‐existing memory. However, previous studies of recall responses to specific antigens suggest a more complex situation, in which older memory is likely to decline if not boosted by repeat exposure or nonspecific heterologous stimulation [52, 53, 54]. Moreover, it has been shown that in some individuals responses to latent viruses, CMV in particular, may account for such a large fraction of T‐cell memory that it impairs other responses [41, 55]. However, in our data, the patterns of T‐cell subset distributions seemed largely independent of the CMV serostatus of the donors, as shown by the fact that in the parameters analyzed, the CMV‐positive individuals were mostly located on the same curves as the CMV‐negative donors. Interestingly, the frequency of SCM cells has also been suggested to remain unaffected by chronic CMV infection [49]. Consistent with this, our analysis of TCR repertoire in SCM subset showed low clonality in both of the analyzed donors, though one of them was CMV‐positive. In general, the clonality index was very similar in the two donors in all populations, except in CD8^+^ EMRA where the CMV‐positive donor displayed higher clonality than the seronegative one. This is consistent with the view that the impact of CMV infection is mainly seen in the generation of end‐stage T‐cell memory. Other factors are likely to contribute to the generation and maintenance of the memory as well, including homeostatic regulation by cytokines [56, 57]. Moreover, it has been suggested that naive T cells may convert to SCM phenotype even in the absence of infection [58].

Finally, the marked tissue‐specific heterogeneity in the distribution and maintenance of T‐cell subsets, shown both by our data and previously by those of others [36, 46], is worth noting. Little of this heterogeneity and dynamic change is revealed by analyzing circulating cells alone.

## Materials and methods

### Donors

Blood samples were obtained from 12 immunologically healthy children (aged from 5 days to 15 years, 6/12 female) undergoing corrective surgery for congenital heart defects and three healthy adults (aged 21–29 years, 1/3 female; Supporting Information Table S1). Written informed consent was obtained from the legal guardians of the children and from the adults. Tissue samples and blood samples were obtained from 13 organ donors (aged 20–67 years, 4/13 female) with no known immunological abnormalities or a cause of death related to infection (Supporting Information Table S2). The cytomegalovirus (CMV) seropositivity of the organ donors and adult blood donors was determined with a clinically accredited ELISA assay (HUSLAB Clinical Microbiology, HUS Diagnostic Center, University of Helsinki and Helsinki University Hospital). Altogether 9 of 16 adult donors were CMV seropositive (Supporting Information Tables S1 and S2). The samples from organ donors were collected in compliance with the Finnish legislation on organ removal and sampling. The study was conducted following the principles of the Declaration of Helsinki and the study was accepted by the Ethics Committee of the Hospital District of Helsinki and Uusimaa (register number HUS/747/2019).

### Sample preparation

Blood samples were drawn to EDTA or lithium‐heparin tubes. Peripheral blood mononuclear cells (PBMCs) were isolated with Ficoll Paque Plus (GE Healthcare, USA) gradient centrifugation. Tissue samples from organ donors (the spleen and the ileum with its mesenteric fat and LNs) were kept moist with physiological saline solution and stored in +4°C. The samples were collected within 2–6 h after their resection and the lymphocytes were extracted immediately. The spleen was homogenized using a 40 μm filter and a plunger and the lymphocytes were separated with Ficoll Paque Plus gradient centrifugation. The LNs were visually identified within the mesenteric fat of the ileal samples, removed with scissors, and the lymphocytes were mechanically extracted with a 40 μm filter and a plunger. To extract the epithelial and lamina propria lymphocytes from ileum, the mucosal surface of ileum was rinsed with PBS and cut with scissors into small pieces that were incubated in 5–10 mL of enzyme solution (RPMI containing 15 mM HEPES, 0.25 mg/mL of DNAse I, and 0.25 mg/mL of collagenase II) on a magnetic shaker in +37°C water bath for 20–30 min. The tissue digest was filtered first with a metallic sieve, then washed with cold PBS containing 10% of FCS and then filtered with 100 μm filter and lastly washed with a cold staining buffer (PBS containing 2% FCS and 2 mM EDTA). Isolated cells were immediately analyzed or sorted with a flow cytometer.

### Flow cytometry

For flow cytometric analysis 1–3 million cells were washed with staining buffer. For staining of surface antigens, cells were incubated 30 min in +4°C with anti‐human fluorescent‐labeled antibodies (CD8‐PE; clone MEM‐31, CD14‐FITC; clone 18D11, CD19‐FITC; clone Hi19a, and CD27‐APC; clone LT27 from Immunotools, Germany, and CD3‐APC‐H7; clone SK7, CD4‐BV510; clone SK3, CD21‐BV650; clone B‐ly4, CD28‐AlexaFluor700; clone CD28.2, CD31‐PeCy7; clone WM59, CD45RA‐BV711; clone HI100, CD95(FAS)‐BV421; clone DX2, CCR7‐PeCF594; clone 150503 from BD Biosciences, USA) and with Live/dead Fixable Green Dead Cell Stain (ThermoFisher Scientific, USA) that were diluted in Brilliant Stain Buffer (BD Biosciences). After subsequent wash with staining buffer, cells were permeabilized with FoxP3 transcription factor staining kit according to the manufacturer's instructions (eBiosciences, USA) for staining of Ki67‐BV786 (clone B56, BD Biosciences). Optimal concentration for antibodies was titrated with live PBMCs. MIATA guidelines for T‐cell assays and principles of published guidelines for flow cytometric analysis were followed in the study [59, 60]. The samples were run on LSRII Fortessa instrument (BD Biosciences) and analyzed with FlowJo software (FlowJo, BD Biosciences) utilizing biological negative controls and fluorescence‐minus‐one staining when applicable. Full gating strategy is shown in Supporting Information Fig. S1. The number of CD4^+^ or CD8^+^ T cells analyzed in each sample is shown in Supporting Information Table S1. The T‐cell subsets with the smallest number of cells obtained (less than 100 CD4^+^ or CD8^+^ T cells) were excluded from the downstream analyses.

For flow cytometric sorting of T‐cell subsets, the cells were stained with LIVE/DEAD Fixable Green Dead Cell Stain and fluorescent‐labeled anti‐human antibodies (CCR7‐Pe‐CF594; clone 150503, CD3‐APC‐H7; clone SK7, CD95‐BV421; clone DX2, CD4‐PerCP‐Cy5.5; clone RPA‐T4, CD45RA‐APC; clone HI100, all from BD Biosciences, CD8‐PE; clone MEM‐31 from Immunotools, and CD62L‐AlexaFluor700; clone DREG‐56 from BioLegend, USA). The sorting was performed using BD FACSAria II instrument (BD Biosciences). The sorted populations were defined as CD45RA^+^CCR7^+^CD62L^+^CD95^–^ being naive, CD45RA^–^CCR7^+^CD62L^+^ being CM, CD45RA^–^CCR7^–^CD62L^–^ being EM, CD45RA^+^CCR7^–^CD62L^–^ being EMRA, and CD45RA^+^CCR7^+^CD62L^+^CD95^+^ being SCM. Sorting strategy is shown in Supporting Information Fig. S7. Sorted cells were stored as dry pellets in –70°C before DNA extraction and TCR sequencing.

### TCR sequencing

The DNA was extracted from sorted cells using the DNeasy kit (Qiagen, Germany) according to the manufacturer's instructions. The TCRα sequences were sequenced with immunoSEQ assay (Adaptive Biotechnologies, USA) at the company facilities. The assay is based on a standardized quantity of quality‐controlled DNA, which allows a quantitative estimate of the number of total genomes with rearranged TCR segments in the sample. In brief, the assay uses a multiplex PCR system spanning the CDR3α region at a length that is sufficient to cover the rearranged V and J segments. Then the amplicon sequencing is performed on the Illumina platform. The gene segment annotations were obtained from IMGT database (www.imgt.org). The PCR and sequencing errors were managed using a synthetic TCR repertoire and barcoded spiked‐in templates. In addition, the synthetic TCR repertoire establishes an amplification baseline that permits the template quantitation.

The TCRα sequences were exported from immunoSEQ ANALYZER and analyzed with programming language Python (python.org). The scripts are available on request. The clonality of a repertoire was estimated with Simpson's clonality index, which is the square root of the Simpson's diversity index $D=\frac{\sum_{i=1\;}^{R}\;\;n_{i}\left(n_{i}-1\right)}{N(N-1)}$, where R is the total number of unique clonotypes in the dataset, $n_{i}$ is the number of cells bearing a certain clonotype, and N is the total number of cells in the dataset. In highly diverse samples, the clonality is close to zero, while in more oligoclonal samples, the clonality values are higher. The fraction of unique overlapping TCRα clonotypes between two T‐cell subsets was estimated calculating Jaccard index that is defined as the size of the intersection of two datasets (*A* and *B*) divided by their union $J\left(A,\;B\right)=|\frac{A\;\cap \;B}{A\cup B}|$. For two fully overlapping repertoires, the Jaccard index value is one. As both Jaccard index and clonality values may depend on the sample size and the obtained sequencing depth, the calculations were repeated with randomly resampling the larger repertoires 100 000 times to the number of unique clonotypes of the smallest sample in the comparison. The resampling was performed with the Python random package.

### Statistics

Statistical test calculations were performed with SPSS Statistics 25 (IBM, USA) and GraphPad Prism 8.0 (GraphPad Software, USA). Kruskal–Wallis test was used to compare the means between multiple groups. Spearman correlation was applied to calculate correlations between two parameters. The results were considered statistically significant with *p* < 0.05. As the frequencies of different cell subsets follow nonparametric distribution, aggregate data are shown as median and interquartile range.

## Conflict of interest

The authors declare no potential conflict of interests.

## Ethics approval and patient consent statements

The study was conducted following the principles of the Declaration of Helsinki and the study was accepted by the Ethics Committee of the Hospital District of Helsinki and Uusimaa (register number HUS/747/2019). A written informed consent was obtained from the parents of the pediatric donors. The samples from organ donors were collected in compliance with the Finnish legislation on organ removal and sampling.

## Author contributions

Conceptualization, investigation, and writing the original manuscript: N.H. and I.H. Software, review, and editing the manuscript: S.S. and J.S. Resources, review, and editing the manuscript: H.I. Methodology, review, and editing of the manuscript: E.K. Conceptualization, project administration, and writing the original manuscript: T.P.A.

### Peer review

The peer review history for this article is available at https://publons.com/publon/10.1002/eji.202149465

**Abbreviations**
CM, central memory ; CMV, cytomeggalovirus ; EM: effector memory
EMRA: effector memory RA+
MLN: mesenteric lymph node
RTE: recent thymic emigrant
SCM: stem cell memory ; TCR, T‐cell receptor

## Supporting information

Supporting information

## Data Availability

The data that support the findings of this study are available from the corresponding author upon reasonable request.
