# Supplementary material for: Peripheral differentiation patterns of human T cells
Source: Eur J Immunol. 2022 Mar 30;52(6):882–94. doi: 10.1002/eji.202149465 (PMC9313577; doi:10.1002/eji.202149465)
Supplement: Supplementary file 1 — Supporting information [file EJI-52-882-s001.pdf]

Supplementary material

Supplementary Fig.1 Gating strategy of naïve, SCM, CM, EM, EMRA, CD31+, CD21+ and Ki67+ subsets in CD4+ and CD8+ compartments. Representative plots are for PBMC (A), the lymph node (B), and the spleen (C) from organ donor 1, and for the ileum (D) from organ donor 7. DCM= dead cell marker.

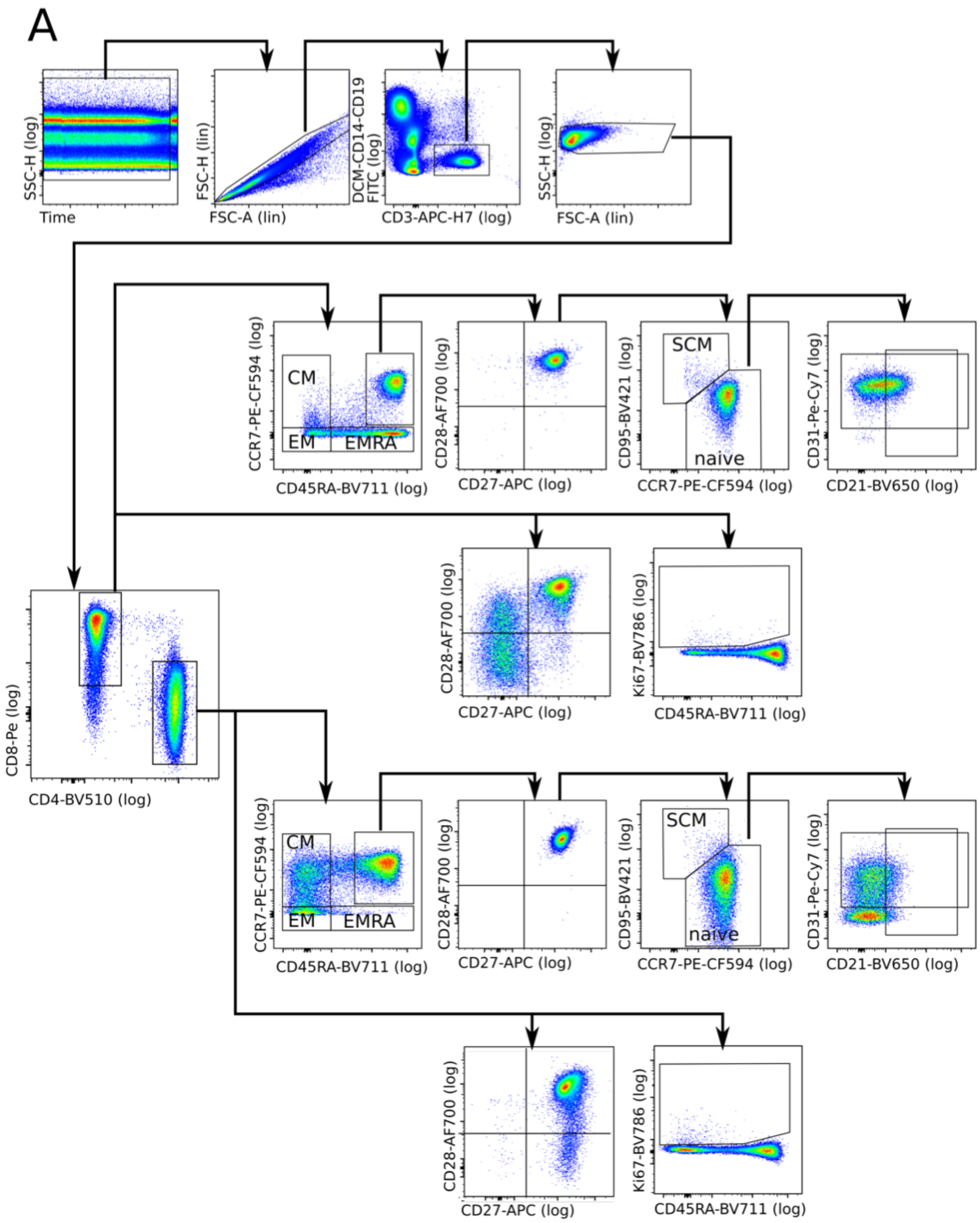

B

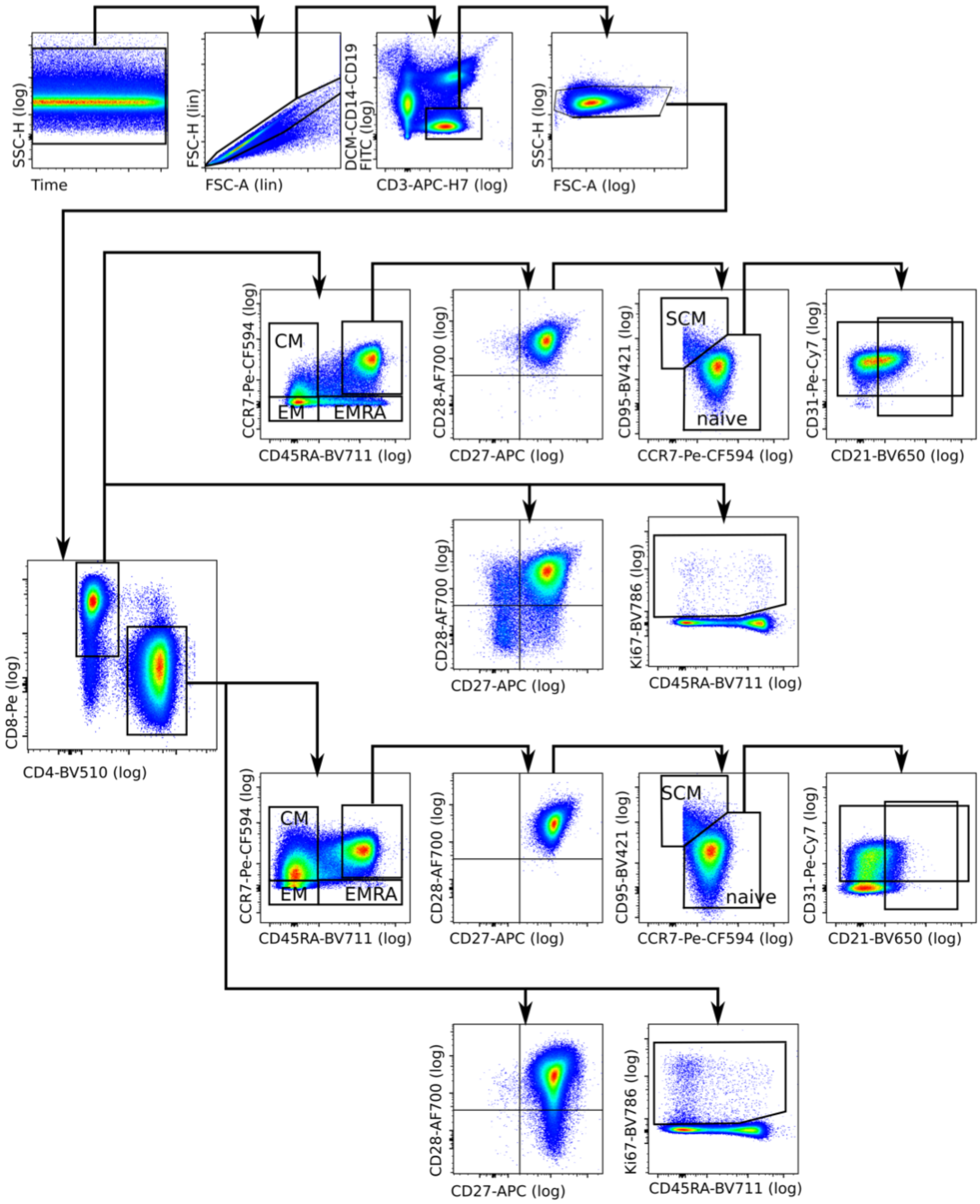
SSC-H (log)
Time
FSC-H (lin)
FSC-A (lin)
DCM-CD14-CD19 FITC (log)
CD3-APC-H7 (log)
SSC-H (log)
FSC-A (log)
CD8-Pe (log)
CD4-BV510 (log)
CCR7-Pe-CF594 (log)
CD45RA-BV711 (log)
CM
EM
EMRA
CD28-AF700 (log)
CD27-APC (log)
CD95-BV421 (log)
CCR7-Pe-CF594 (log)
SCM
naive
CD31-Pe-Cy7 (log)
CD21-BV650 (log)
Ki67-BV786 (log)
CD45RA-BV711 (log)

C

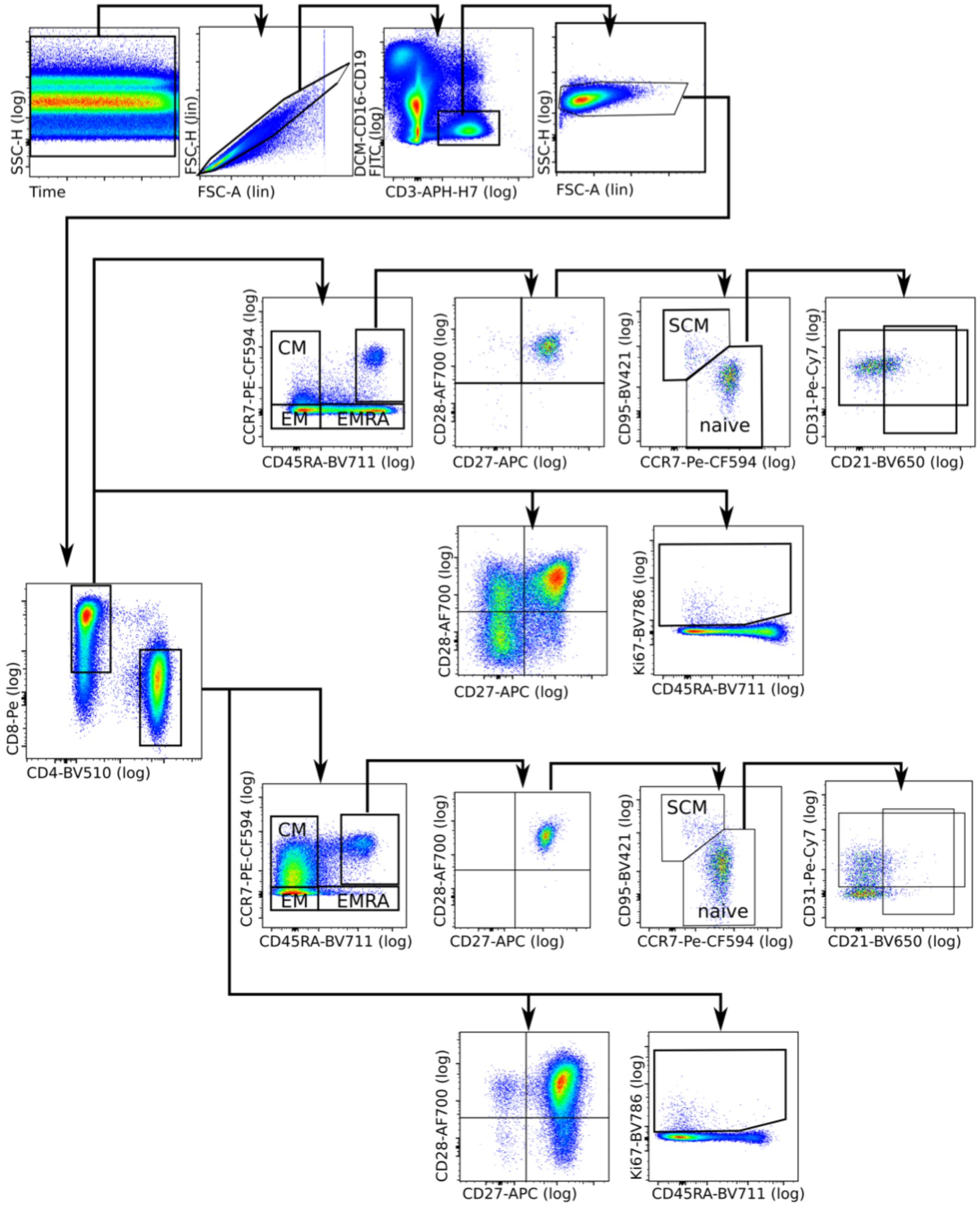

SSC-H (log)
Time
FSC-H (lin)
FSC-A (lin)
DCM-CD16-CD19 FITC (log)
CD3-APH-H7 (log)
SSC-H (log)
FSC-A (lin)
CCR7-PE-CF594 (log)
CM
EM
EMRA
CD45RA-BV711 (log)
CD28-AF700 (log)
CD27-APC (log)
CD95-BV421 (log)
SCM
naive
CCR7-Pe-CF594 (log)
CD31-Pe-Cy7 (log)
CD21-BV650 (log)
Ki67-BV786 (log)
CD8-Pe (log)
CD4-BV510 (log)

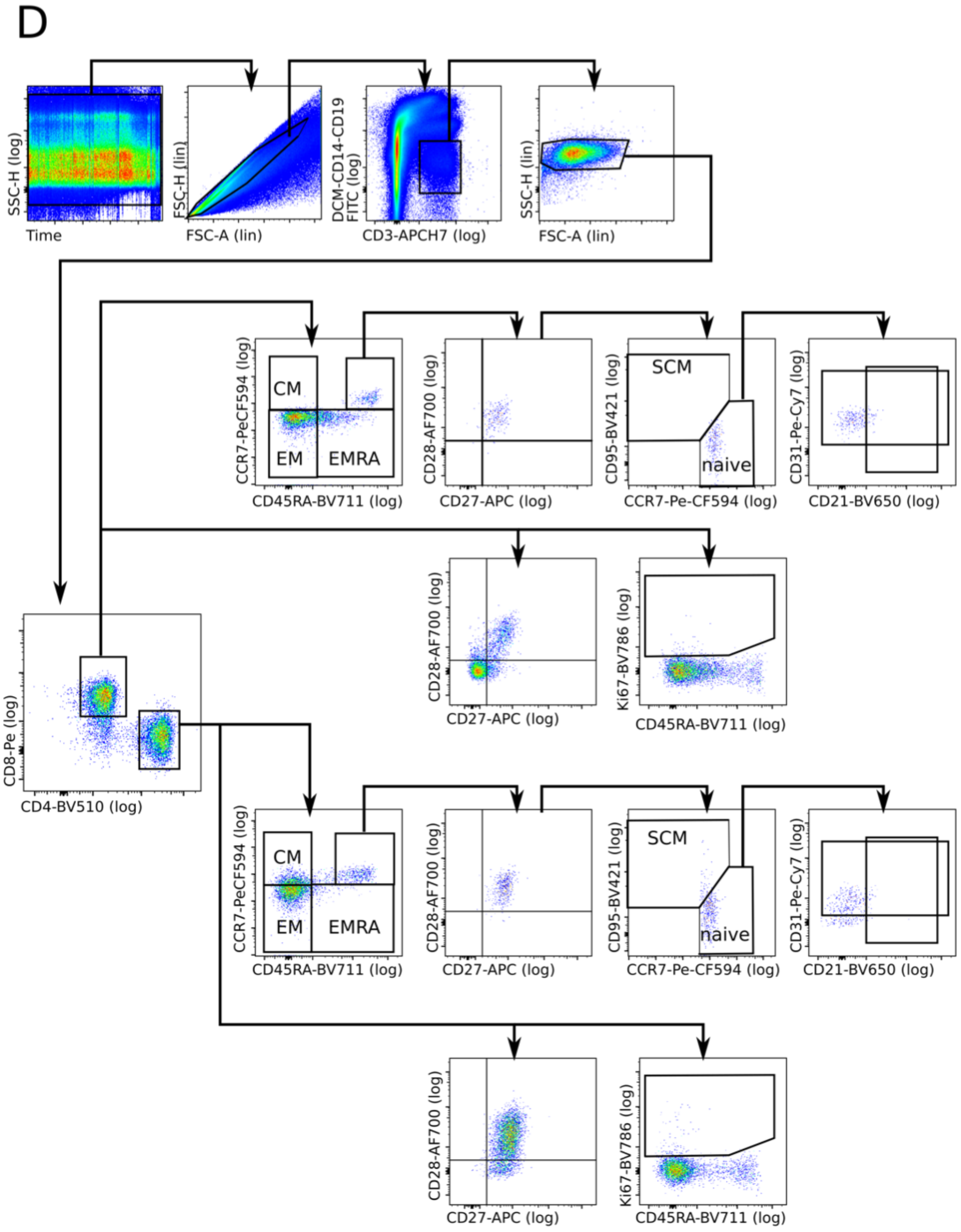
D
SSC-H (log)
Time
FSC-H (lin)
FSC-A (lin)
DCM-CD14-CD19 FITC (log)
CD3-APCH7 (log)
SSC-H (lin)
FSC-A (lin)
CCR7-PeCF594 (log)
CD45RA-BV711 (log)
CM
EM
EMRA
CD28-AF700 (log)
CD27-APC (log)
CD95-BV421 (log)
CCR7-Pe-CF594 (log)
SCM
naive
CD31-Pe-Cy7 (log)
CD21-BV650 (log)
Ki67-BV786 (log)
CD8-Pe (log)
CD4-BV510 (log)

Supplementary Fig. 2 Frequencies of CD21+ naïve cells within CD4+ (A) and CD8+ (B) T-cell compartments in PBMC, MLN, spleen and ileum.

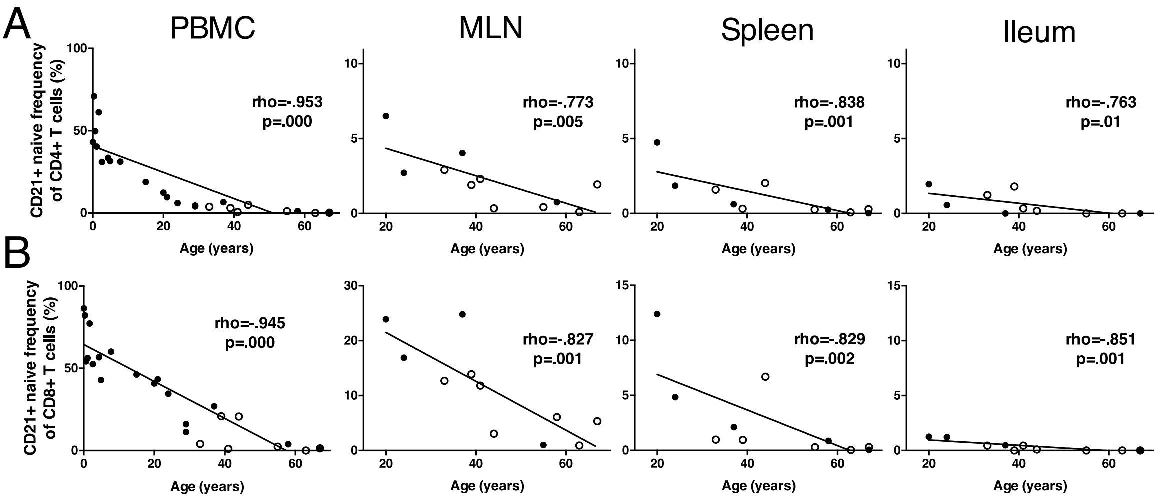

Supplementary Fig. 3 The expression of CD27 and CD28 in CD4+ and CD8+ compartments among all T cells and among the EM (CD45RA-CCR7-) subset in blood (B), spleen (S), lymph node (L) and ileum (I).

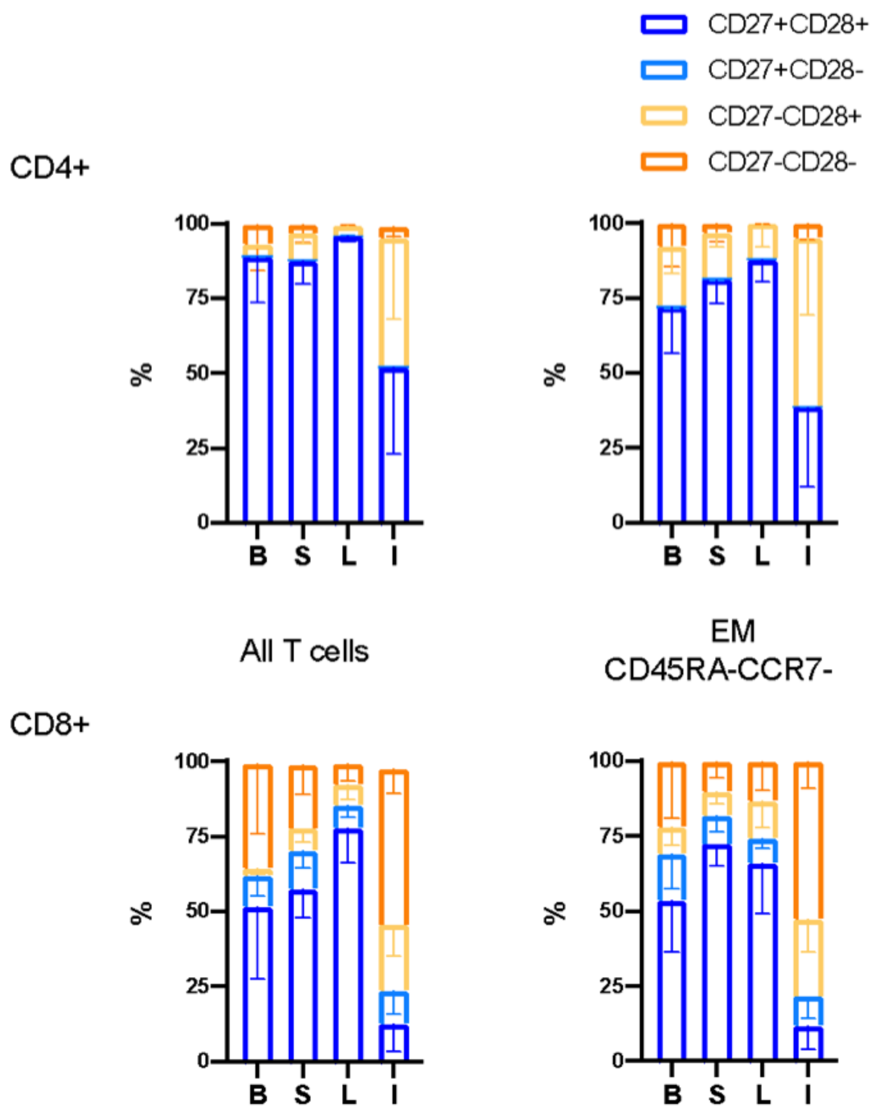

Supplementary Fig. 4 Frequencies of naïve, CM, EM and EMRA subsets in blood, MLN, spleen and ileum at individual level in CD4+ compartment (n=7) and in CD8+ compartment (n=8). Each color indicates different individual.

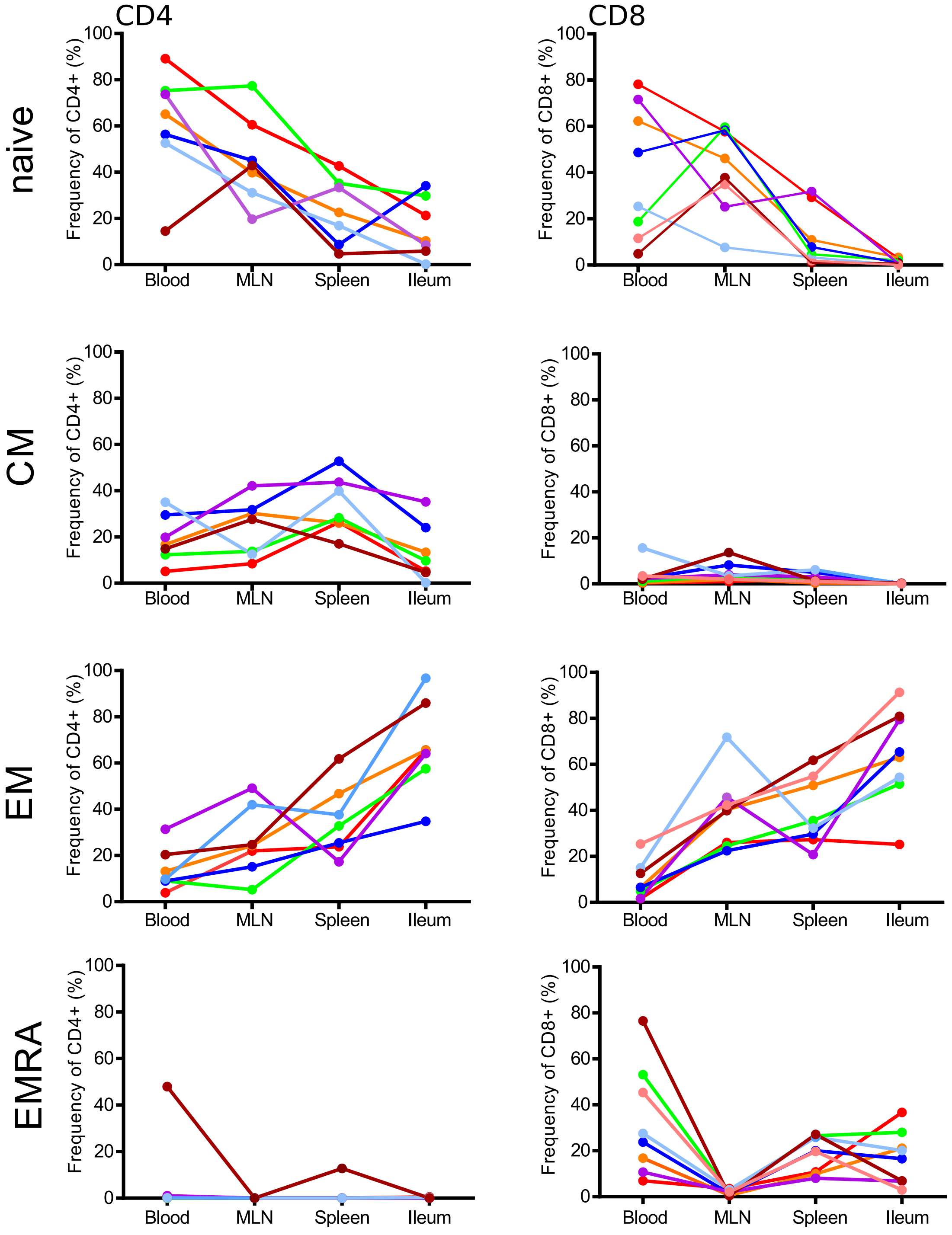

Supplementary Fig. 5 Fraction of Ki67+ cells in naïve, CM, EM and EMRA subsets in blood, MLN, spleen and ileum at individual level in CD4+ compartment (n=7) and in CD8+ compartment (n=8). Each color indicates different individual.

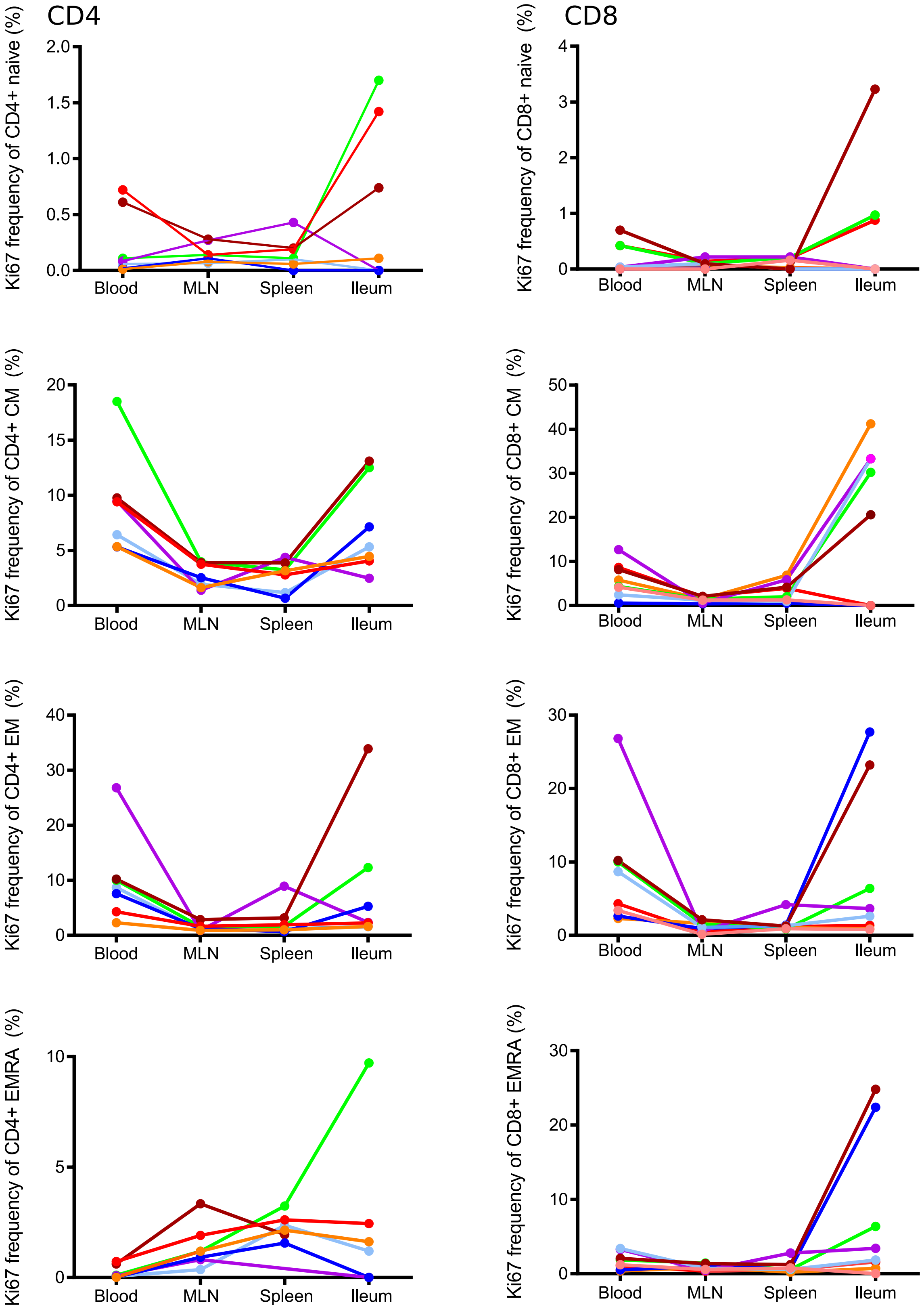

Supplementary Fig. 6 Expression of Ki67 in different memory subsets in different tissues in the age groups: 20–35 years, 36–45 years and >45 years. For age group 20–35 years n=3 for all tissues; for age group 36–45 years n=4 except for spleen where n=3; for age group >45 years n=5 for blood, n=4 for MLN, n=5 for spleen and n=3 ileum.

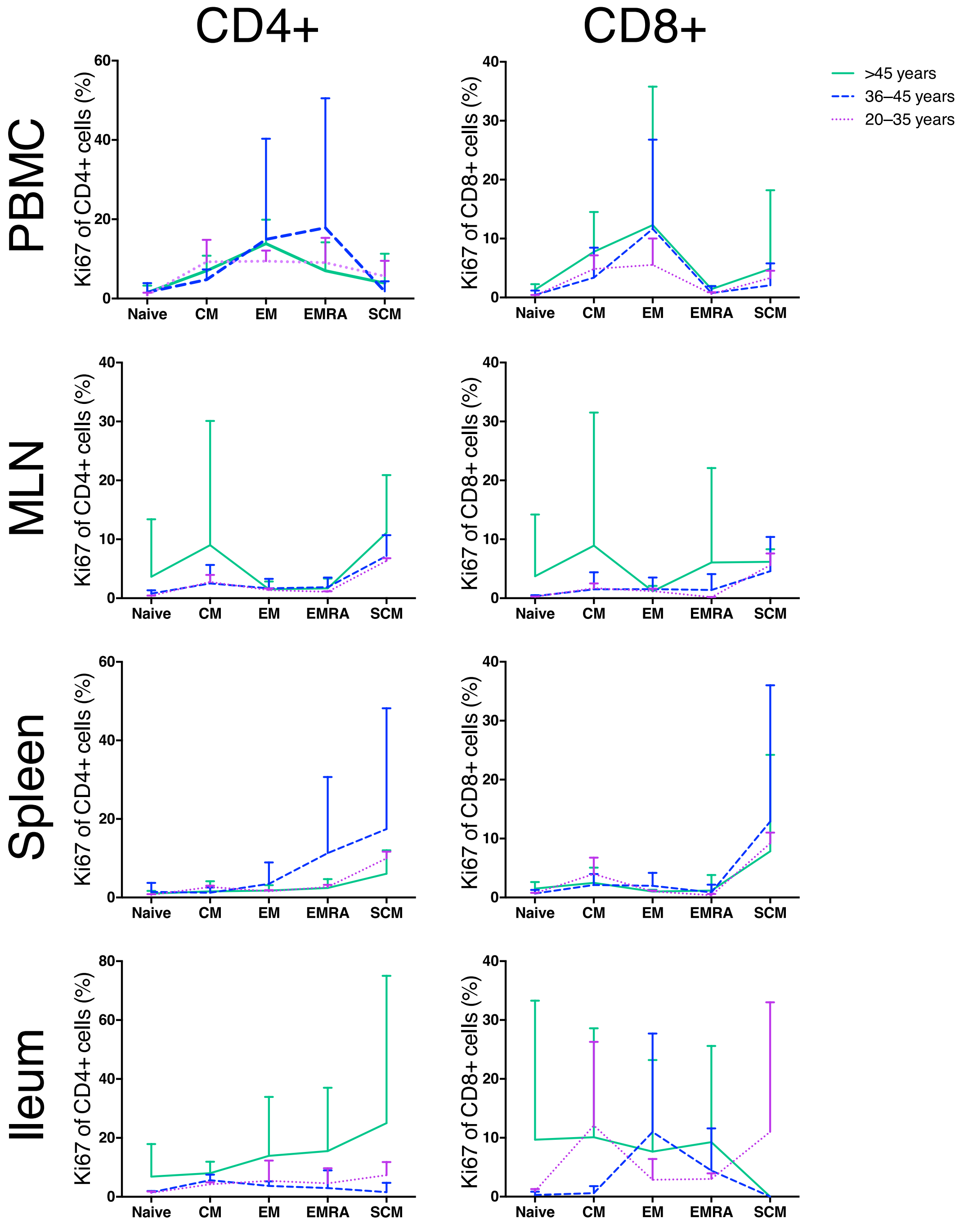

Supplementary Fig. 7 Gating strategy to sort naïve, SCM, CM, and EM subsets in the CD4+ compartment, and naïve, SCM, EM and EMRA subsets in the CD8+ T-cell compartment.

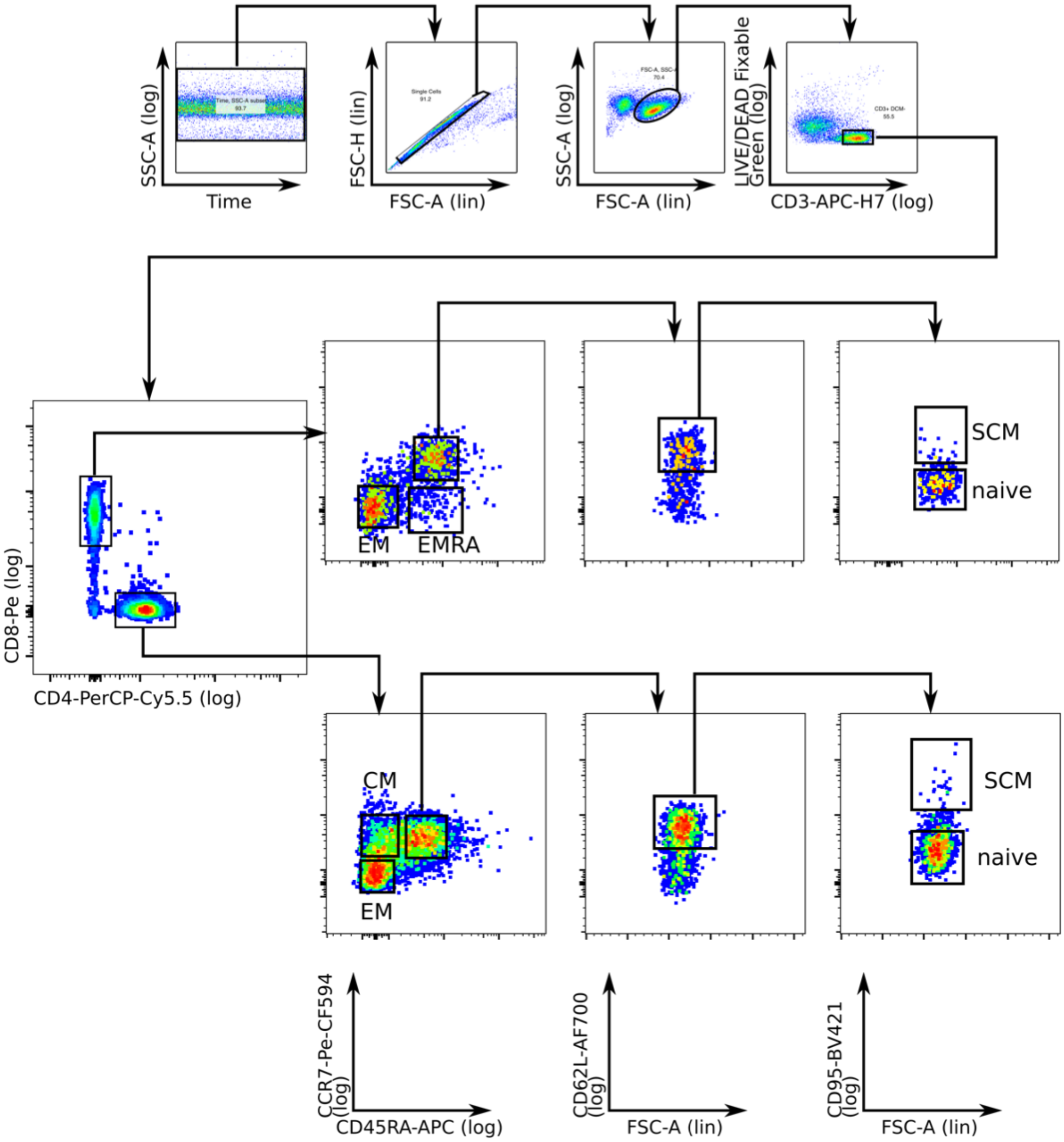

Supplementary table 1. Details of blood donors.

				Absolute counts of	
**Donor**	**Sex**	**Age**	**CMV**	**CD4+**	**CD8+**
Blood donor 1	female	2.6 years	NA	83 092	27 995
Blood donor 2	female	15 years	NA	79 574	52 839
Blood donor 3	female	1.1 years	NA	101 988	46 347
Blood donor 4	male	4.3 years	NA	104 702	48 568
Blood donor 5	male	5 days	NA	46 616	20 177
Blood donor 6	male	7.8 years	NA	116 699	69 608
Blood donor 7	male	1.7 years	NA	196 269	133 379
Blood donor 8	male	8.2 months	NA	497 000	127 209
Blood donor 9	female	6 days	NA	79 798	47 167
Blood donor 10	male	17 days	NA	183 321	58 402
Blood donor 11	female	4.9 years	NA	80 724	41 423
Blood donor 12	female	4.6 months	NA	979 000	183 665
Blood donor 13	male	21 years	negative	109 636	73 893
Blood donor 14	female	29 year	positive	76 595	44 903
Blood donor 15	male	29 years	negative	315 276	158 049

Supplementary table 2. Details of organ donors.

Donor	Sex	Age	CMV	Cause of death	Analyses	Tissues	PBMC	MLN	Spleen	Ileum	PBMC	MLN	Spleen	Ileum
Organ donor 1	male	37 years	negative	subarachnoid hemorrhage	Flow cytometry, cell sorting and TCRα sequencing	blood, MLN, spleen, ileum	41 349	363 534	76 043	<100 CD4+ T cells, not analyzed downstream	36 267	131 153	56 153	418
Organ donor 2	female	35 years	positive	head trauma	Cell sorting and TCRα sequencing	MLN	No flow cytometric analysis							
Organ donor 3	female	67 years	negative	subarachnoid hemorrhage		blood, spleen, ileum	15 815	NA	134 672	6 086	4 333	NA	30 799	7 762
Organ donor 4	male	58 years	negative	subarachnoid hemorrhage		blood, MLN, spleen, ileum	11 297	91 951	80 990	<100 CD4+ T cells, not analyzed downstream	8 373	18 152	65 596	<100 CD8+ T cells, not analyzed downstream
Organ donor 5	male	41 years	positive	cerebral infarction		blood, MLN, ileum	1 767	98 280	NA	10 672	3 188	52 204	NA	17 298
Organ donor 6	male	67 years	positive	intracerebral hemorrhage		blood, MLN, spleen, ileum	3 910	22 971	121 438	<100 CD4+ T cells, not analyzed downstream	3 094	3 473	153 068	136
Organ donor 7	male	24 years	negative	anoxia		blood, MLN, spleen, ileum	37 612	722 000	107 584	14 529	32 906	297 430	122 151	18 507
Organ donor 8	male	20 years	negative	anoxia	Flow cytometry	blood, MLN, spleen, ileum	66 847	736 000	268 225	3 169	56 547	546 000	169 099	21 696
Organ donor 9	male	39 years	positive	head trauma		blood, MLN, spleen, ileum	82 682	1 070 000	115 346	167	39 672	204 211	90 714	<100 CD8+ T cells, not analyzed downstream
Organ donor 10	female	63 years	positive	subarachnoid hemorrhage		blood, MLN, spleen, ileum	16 709	432 000	82 566	3 147	15 464	153 654	103 522	5 457
Organ donor 11	male	44 years	positive	intracerebral hemorrhage		blood, MLN, spleen, ileum	17 151	97 458	125 260	2 722	5 762	25 643	34 670	6 035
Organ donor 12	male	55 years	positive	head trauma		blood, MLN, spleen, ileum	52 348	642 000	281 653	30 641	34 360	128 137	146 897	137 539
Organ donor 13	male	33 years	positive	subarachnoid hemorrhage		blood, MLN, spleen, ileum	22 560	375 526	173 686	6 480	28 055	98 424	280 834	18 722
